# Long-Term Effectiveness and Cost-Effectiveness of Videoconference-Delivered Cognitive Behavioral Therapy for Obsessive-Compulsive Disorder, Panic Disorder, and Social Anxiety Disorder in Japan: One-Year Follow-Up of a Single-Arm Trial

**DOI:** 10.2196/17157

**Published:** 2020-04-23

**Authors:** Kazuki Matsumoto, Sayo Hamatani, Kazue Nagai, Chihiro Sutoh, Akiko Nakagawa, Eiji Shimizu

**Affiliations:** 1 Research Center for Child Mental Development Chiba University Chiba Japan; 2 Japan Society for the Promotion of Science Tokyo Japan; 3 Research and Education Center of Health Sciences Graduate School of Health Sciences Gunma University Gunma Japan; 4 Department of Cognitive Behavioral Physiology Graduate School of Medicine Chiba University Chia Japan

**Keywords:** long-term effectiveness, cost-effectiveness, videoconference-delivered cognitive behavioral therapy, internet-based cognitive behavioral therapy, obsessive-compulsive disorder, panic disorder, social anxiety disorder

## Abstract

**Background:**

Face-to-face individual cognitive behavioral therapy (CBT) and internet-based CBT (ICBT) without videoconferencing are known to have long-term effectiveness for obsessive-compulsive disorder (OCD), panic disorder (PD), and social anxiety disorder (SAD). However, videoconference-delivered CBT (VCBT) has not been investigated regarding its long-term effectiveness and cost-effectiveness.

**Objective:**

The purpose of this study was to investigate the long-term effectiveness and cost-effectiveness of VCBT for patients with OCD, PD, or SAD in Japan via a 1-year follow-up to our previous 16-week single-arm study.

**Methods:**

Written informed consent was obtained from 25 of 29 eligible patients with OCD, PD, and SAD who had completed VCBT in our clinical trial. Participants were assessed at baseline, end of treatment, and at the follow-up end points of 3, 6, and 12 months. Outcomes were the Yale-Brown Obsessive-Compulsive Scale (Y-BOCS), Panic Disorder Severity Scale (PDSS), Liebowitz Social Anxiety Scale (LSAS), Patient Health Questionnaire–9 (PHQ-9), General Anxiety Disorder–7 (GAD-7), and EuroQol-5D-5L (EQ-5D-5L). To analyze long-term effectiveness, we used mixed-model analysis of variance. To analyze cost-effectiveness, we employed relevant public data and derived data on VCBT implementation costs from Japanese national health insurance data.

**Results:**

Four males and 21 females with an average age of 35.1 (SD 8.6) years participated in the 1-year follow-up study. Principal diagnoses were OCD (n=10), PD (n=7), and SAD (n=8). The change at 12 months on the Y-BOCS was −4.1 (*F*_1_=4.45, *P*=.04), the change in PDSS was −4.4 (*F*_1_=6.83, *P*=.001), and the change in LSAS was −30.9 (*F*_1_=6.73, *P*=.01). The change in the PHQ-9 at 12 months was −2.7 (*F*_1_=7.72, *P*=.007), and the change in the GAD-7 was −3.0 (*F*_1_=7.09, *P*=.009). QALY at 12 months was 0.7469 (SE 0.0353, 95% Cl 0.6728-0.821), and the change was a significant increase of 0.0379 (*P*=.01). Total costs to provide the VCBT were ¥60,800 to ¥81,960 per patient. The set threshold was ¥189,500 ($1723, €1579, and £1354) calculated based on willingness to pay in Japan.

**Conclusions:**

VCBT was a cost-effective way to effectively treat Japanese patients with OCD, PD, or SAD.

**Trial Registration:**

University Hospital Medical Information Network Clinical Trials Registry UMIN000026609; https://upload.umin.ac.jp/cgi-open-bin/ctr_e/ctr_view.cgi?recptno=R000030495

## Introduction

### Background

Obsessive-compulsive disorder (OCD), panic disorder (PD), and social anxiety disorder (SAD) are mental health illnesses that create severe obstacles for patients in their daily lives [1]. The long-term effectiveness of treatment is worth evaluating, because OCD, PD, and SAD often recur even after improvement following treatment [2-4]. In particular, it is important to guide effective health care policy in countries such as Japan, which have instituted universal public health care insurance systems [5], to optimize limited resources and maintain medical services in consideration of cost-effectiveness.

Telepsychiatry can be delivered to established therapy patients in developed countries where there is wide availability of information and communication devices and internet use is high. Within telepsychiatry, videoconference-delivered cognitive behavioral therapy (VCBT) has proved promising, with the potential to improve the accessibility of specialized care to patients with OCD, PD, and SAD [6]. Even with simple Web cameras, the internet, and information and communication equipment, psychiatrists can significantly improve symptoms by properly examining patients with mental illness, delivering psychological education, and dispensing medication [7]. Multiple clinical trials have reported significant reductions in symptoms of depression, OCD, PD, and SAD as a result of VCBT [6,8,9]. However, we know little about the long-lasting (12 or more months) effectiveness and cost-effectiveness of VCBT, despite its proven short-term effectiveness [4,9,10].

VCBT requires a videoconferencing system, thereby making it more expensive compared with face-to-face cognitive behavioral therapy (CBT). For facilities that provide health care services, VCBT is a little more expensive than traditional CBT. However, for patients, VCBT is less burdensome than face-to-face CBT, as there are no travel costs or time costs associated with hospital visits. VCBT puts the burden of cost on the facility; thus, it is particularly important to assess whether its adoption is a worthwhile approach from the perspective of efficient health care policy.

### Objectives of the Study

This study’s main objectives were to assess the long-term effectiveness of VCBT for patients with OCD, PD, or SAD and estimate its cost-effectiveness in Japan.

## Methods

### Study Design

In this study, we included data from our previous clinical trials and follow-ups [6]. We obtained written consent from participants in two stages. First, we obtained participants’ written consent forms to research feasibility of VCBT at face-to-face screening before the intervention. Second, those who consented to participate in the follow-up study were requested to resend signed consent forms provided to the researchers. The questionnaires on symptomology were sent by mail or the data collected telephonically at 3, 6, 8, and 12 months after the end of VCBT. These data were used in this study.

In March 2018, the Cognitive Behavioral Therapy Center at Chiba University Hospital implemented a prospective observational study involving all patients who participated in VCBT (reference number: G28038, UMIN000026609) [6]. The study was registered with University Hospital Medical Information Network Clinical Trials Registry [UMIN000026609]. In a follow-up study after the intervention, the institutional review board of Chiba University approved the study protocol (No. 3048).

### Participants and Eligibility Criteria in the Clinical Trial

All participants had received face-to-face treatment from the attending physician (psychiatrist) during a previous clinical trial period [6]. VCBT was provided in addition to ongoing face-to-face treatment including pharmacotherapy. Inclusion criteria for our previous clinical trial included informed consent to participate in the study; having a primary diagnosis of OCD, PD, or SAD based on the Mini-International Neuropsychiatric Interview [11,12]; being aged between 19 and 65 years; and having access to the internet at home [6].

### Outcomes

#### Symptomatology

The following Japanese version of three scales were used to assess the severity of the three disorders. The Yale-Brown Obsessive-Compulsive Scale (Y-BOCS) was used to measure OCD by identifying the patient’ contents of obsessions and compulsions on the symptom checklist and assessing their severity in 4 stages using responses to 10 items on the symptom severity scale [13,14]. The Panic Disorder Severity Scale (PDSS) was used when PD was the principal diagnosis [15,16]. PDSS is a 7-item questionnaire on frequency of panic attacks, extent of subjective distress, impact on daily life, and so on, with response options ranging from 0 to 4 in severity. The Liebowitz Social Anxiety Scale (LSAS) was used for participants whose principal diagnosis was SAD [17,18]. LSAS is a 24-item questionnaire intended to evaluate the extent of anxiety and avoidance in social situations where social anxiety is noticeable (eg, public speaking, talking to strangers).

We also assessed depression and general anxiety associated using responses to the Patient Health Questionnaire–9 (PHQ-9) and Generalized Anxiety Disorder 7 (GAD-7). PHQ-9 has 9 questions related to depression status set [19,20], and GAD-7 is a 7-question instrument about general anxiety [21]. We evaluated the quality-adjusted life year (QALY) calculation in the EuroQol 5-Dimension 5-Level (EQ-5D-5L) instrument to assess the cost-effectiveness of the VCBT as a health care technology [22]. The EQ-5D-5L questions determine quality of life [22,23]. Health status is determined in five dimensions: degree of movement, personal management, normal activity, pain/discomfort, and anxiety/hiding.

#### Criteria Used to Define Therapeutic Response and Remission

To calculate responsiveness to VCBT treatment and remission rates after the VCBT, we used criteria employed by previous studies regarding the severity rating scales of the three disorders (Y-BOCS, PDSS, and LSAS). Regarding OCD, treatment response was defined as a 35% or greater reduction in the total Y-BOCS score, and remission was defined as a 12-month Y-BOCS≤14 [24]. Regarding PD, treatment response was defined as a 40% or greater reduction in total PDSS score, and remission was defined as a 12-month PDSS≤ 7 [25]. For SAD, treatment response was defined as a 31% or greater reduction in total LSAS score, and remission was defined as a 12-month LSAS≤35 [17].

#### Sources for Cost-Effectiveness

We calculated the total VCBT cost using the sum of the costs of implementing the intervention: (1) health care costs (¥3500-¥4800 × 16 sessions) and (2) costs of videoconferencing (license fee ¥1490 per month × 4 months in Webex (Cisco), ¥300 × 16 sessions in curon (MICIN, Inc) [26,27]. Note that in Japan, the cost of CBT in health care settings differs depending on whether it is performed by a doctor or jointly performed by a doctor and a nurse [28]. The cost-effectiveness threshold of the VCBT intervention was based on the willingness-to-pay (WTP) figure determined in a previous study (¥5 million) [29].

We did not assume that hardware would have to be newly purchased in order to access VCBT. This was because, as reported by the Ministry of Internal Affairs and Communications in 2017, ownership of information communication equipment in Japan was at 94.8% for mobile devices in general and 72.5% for PCs and because the penetration rate of information and communication equipment and the internet was at more than 80.9% for all households [30].

### Statistical Analyses

Statistical analysis and reporting were performed in accord with the CONSORT-EHEALTH guidelines [31]. All statistical analyses were described in the statistical analysis plan, which was fixed before the database lock. All efficacy analyses were primarily based on the entire analytical dataset. Summary statistics were generated on all baseline variables with frequencies and proportions calculated on categorical data and means and standard deviations calculated on continuous variables.

The main analysis compared the baseline assessment scores with those obtained at the 12-month posttreatment follow-up. The differences were estimated using mixed-model analysis of variance (ANOVA) on all patients displaying symptoms in each scale (Y-BOCS, PDSS, LSAS, PHQ-9, and GAD-7), taking into account missing values, individual variance, and multiple measurement points.

Analysis of secondary outcomes was performed in an identical fashion to that of the primary analysis. To analyze cost-effectiveness using the EQ-5D-5L, QALY scores were estimated via area-under-the-curve analysis, which involved summing the areas of the distribution shapes for utility scores over the study period [22]. We calculated QALY summary statistics using the EQ-5D-5L data during the follow-up period complemented by multivariate imputation by chained equations (MICE) and last observation carried forward (LOCF). MICE was used as a guide for 100 completions [32].

The amount of change in QALY was calculated from the difference between QALY and the actually observed utility value assuming no change from the utility value of EQ-5D-5L at baseline. We calculated a summary statistic for the change in QALY and performed a paired *t* test. The method for calculating the change in QALY was as follows: QALY change amount = (baseline and end of treatment, 3 months, 6 months, 8 months, or area under the curve connecting the utility values including 12 months) – (effective value at each time point is baseline utility value and area under the curve assuming no change).

Cost-effectiveness of the VCBT was analyzed as follows. The additional consumption of health care resources was divided by the benefits (such as QALY) gained from the health care intervention to calculate an incremental cost-effectiveness ratio (ICER). When the ICER, such as cost per QALY, was less than a predetermined threshold, the intervention was considered cost-effective [33]. These thresholds were: (1) £20,000-£30,000 per QALY at the UK National Institute for Health and Care Excellence (NICE) [34], (2) $62,000 in the United States, and (3) ¥5 million in Japan [29]. The formulae used to calculate cost-effectiveness of VCBT, cost of VCBT, and WTP were as follows:

- Cost-effectiveness of VCBT = WTP − cost of VCBT
- Cost of VCBT = (videoconference system costs) + traditional CBT costs
- WTP = increased QALYs × threshold in Japan (¥5 million)

Calculated cost-effectiveness greater than one indicated that VCBT was a cost-effective intervention. WTP was calculated by multiplying the increase in QALY between baseline and 12-month follow-up after VCBT using the Japanese cost-effectiveness threshold (¥5 million). Incremental cost-effectiveness ratio per QALY was calculated by dividing the increase in QALY between baseline and 12-month follow-up after VCBT using total cost of VCBT.

## Results

### Participants

**Figure 1:**
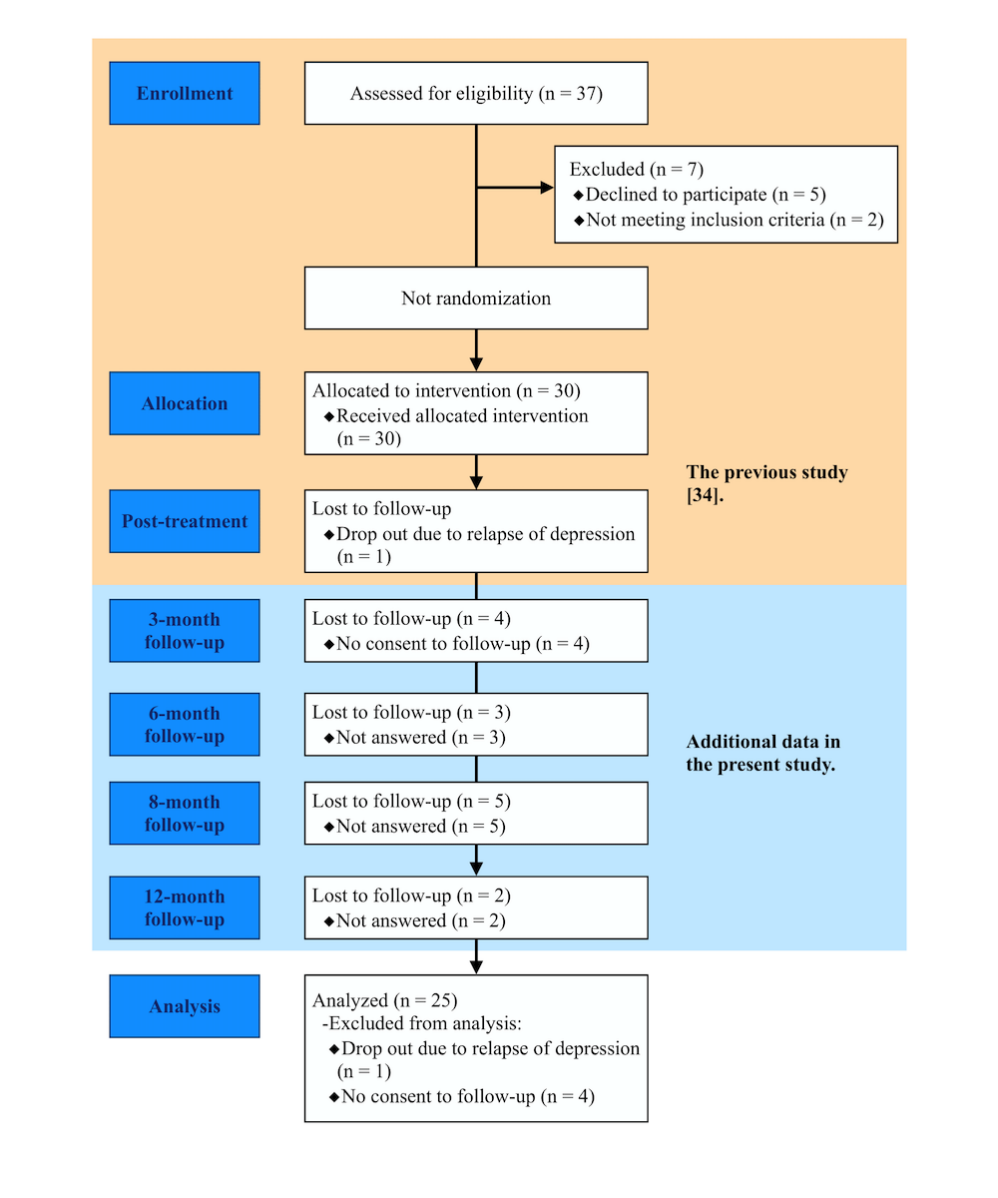
Participant flow.

The sample comprised 4 males and 21 females, aged 20 to 54 years (mean 35.1 [SD 8.6] years) with 12 to 18 years of education (mean 14.72 [SD 1.90] years). Except for their principal diagnoses, participants’ demographic and diagnostic data are described in Table 1, and the sampling procedure is illustrated in Figure 1.

**Table 1:** Participant clinical and demographic characteristics.

Characteristics		Overall (n=25)	OCD^a^ (n=10)	PD^b^ (n=7)	SAD^c^ (n=8)
Age in years, mean (SD)		35.1 (8.6)	37.7 (6.9)	36.1 (9.3)	30.9 (9.4)
Gender (female), n (%)		21 (84)	8 (80)	7 (100)	6 (75)
Employed, n (%)		14 (56)	3 (12)	5 (71)	6 (75)
Pharmacotherapy (yes), n (%)		9 (36)	5 (50)	3 (43)	1 (13)
**Comorbidity, n (%)**					
	Depression	3 (12)	1 (10)	0 (0)	2 (24)
	Panic/agoraphobia	2 (11)	2 (20)	0 (0)	0 (0)
	PTSD^d^	1 (4)	1 (10)	0 (0)	0 (0)
	Alcohol dependence	1 (4)	0 (0)	0 (0)	1 (13)

^a^OCD: obsessive-compulsive disorder.

^b^PD: panic disorder.

^c^SAD: social anxiety disorder.

^d^PTSD: posttraumatic stress disorder.

### Long-Term Effectiveness

**Figure 2:**
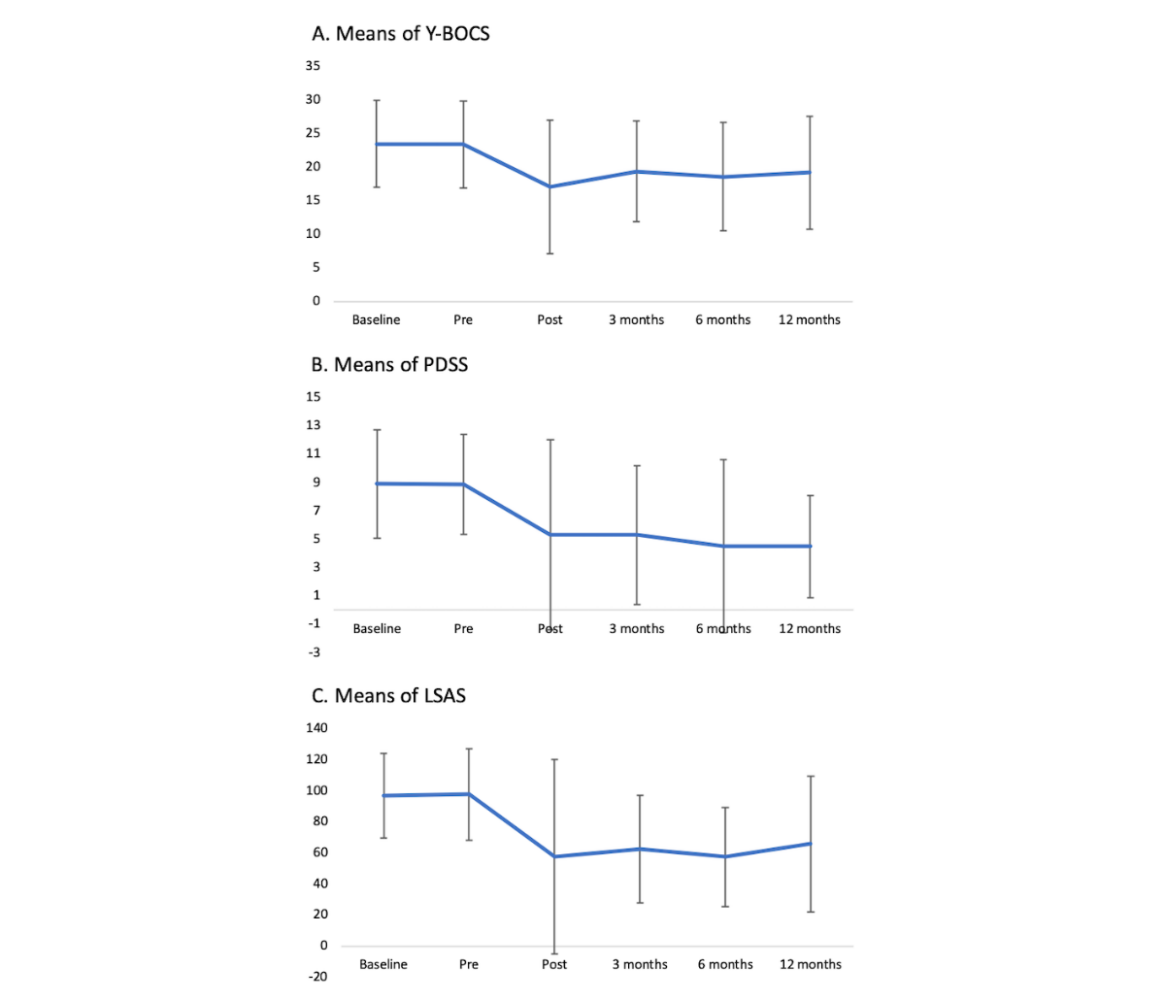
Participants’ changes in symptomology.

Mixed-model ANOVA results regarding the long-term effectiveness of VCBT showed statistically significant improvement in participant symptoms (Table 2 and Figure 2). Changes in total mean scores between baseline and 12-month assessment were –4.1 on the Y-BOCS (*F*_1_=4.45, *P*=.04), –4.4 on the PDSS (*F*_1_=6.83, *P*=.01), and –30.9 on the LSAS (*F*_1_=6.73, *P*=.01). Changes in the total PHQ-9 (depression) and GAD-7 (general anxiety) scores between baseline and 12-month follow-up assessment were –2.7 on the PHQ-9 (*F*_1_=7.72, *P*=.007) and –3.0 on the GAD-7 (*F*_1_= 7.09, *P*=.009).

**Table 2:** Mixed-model analysis of variance results on changes in participant symptomology.

Characteristics		n	Score mean (SD)	Min-max^a^	*P* value
**Y-BOCS^b^**		—	—	—	.04
	Baseline	10	23.3 (6.5)	15 (36)	—
	Posttreatment	10	17.1 (9.9)	2 (34)	—
	3-month	10	19.4 (7.5)	9 (32)	—
	6-month	10	18.6 (8.1)	7 (32)	—
	12-month	10	19.2 (8.4)	8 (29)	—
**PDSS^c^**		—	—	—	.01
	Baseline	7	8.9 (3.8)	5 (16)	—
	Posttreatment	7	5.3 (6.7)	0 (19)	—
	3-month	7	5.4 (4.9)	2 (13)	—
	6-month	6	4.5(6.1)	0 (16)	—
	12-month	6	4.5 (3.6)	0 (10)	—
**LSAS^d^**		—	—	—	.01
	Baseline	8	96.6 (27.3)	53 (132)	—
	Posttreatment	8	57.4 (34.7)	21 (128)	—
	3-month	8	62.6 (34.4)	20 (112)	—
	6-month	6	57.3 (31.9)	7 (85)	—
	12-month	7	65.7 (43.8)	10 (118)	—
**PHQ-9^e^**		—	—	—	.007
	Baseline	25	8.8 (6.2)	0 (23)	—
	Posttreatment	25	6.8 (7.0)	0 (22)	—
	3-month	25	7.2 (5.8)	0 (24)	—
	6-month	22	6.6 (6.1)	0 (19)	—
	12-month	23	6.1 (5.7)	0 (20)	—
**GAD-7^f^**		—	—	—	.009
	Baseline	25	8.8 (5.3)	0 (20)	—
	Posttreatment	25	5.5 (5.1)	0 (16)	—
	3-month	25	7.2 (4.6)	0 (19)	—
	6-month	25	6.3 (5.0)	0 (21)	—
	12-month	23	5.8 (4.5)	0 (14)	—

^a^min-max: minimum to maximum.

^b^Y-BOCS: Yale-Brown Obsessive-Compulsive Scale.

^c^PDSS: Panic Disorder Severity Scale.

^d^LSAS: Livobitz Social Anxiety Scale.

^e^PHQ-9: Patient Health Questionnaire–9.

^f^GAD-7: Generalized Anxiety Disorder–7.

To investigate the predictive effects that symptoms of patients’ depression at pretreatment may have had on the treatment response change posttreatment, multiple regression analyses in simultaneous forced entry were performed. The treatment response percentage change was set as a dependent variable in multiple regression analyses. We set depressive symptoms due to PHQ-9 as independent variables. The treatment response percentage change was calculated by dividing the total baseline score with the score difference between baseline and 12-month. The treatment response percentage change in this study was the decline in baseline Y-BOCS, PDSS, or LSAS score.

The degree of change (in percentages) in the treatment response was analyzed as a continuous variable. Statistical analysis was performed using SPSS Statistics version 24.00 (IBM Corp). Multiple regression analysis showed that the effects of depression on therapeutic response rates were not significant across the data (*β*=–1.74, adjusted *R*^2^=.13, SE 25.29, *P*=.053, VIF (variance inflation factor)=1.00), OCD (*β*=–1.60, adjusted *R*^2^=.24, SE 19.46, *P*=.16), PD (*β*=–0.41, adjusted *R*^2^=.25, SE 38.82, *P*=.96), and SAD (*β*=–0.73, adjusted *R*^2^=.18, SE 24.02, *P*=.77).

### Therapeutic Response and Remission Rates

At the 12-month follow-up assessment, treatment response rate was 32% (8/25) and remission rate was 40% (10/25; Table 3).

**Table 3:** Participant response and remission rates at each follow-up end point.

Characteristics		Overall (n=25), n (%)	OCD^a^ (n=10), n (%)	PD^b^ (n=7), n (%)	SAD^c^ (n=8), n (%)
**Response**					
	Posttreatment	12 (48)	4 (40)	4 (57)	4 (50)
	3-month	10 (40)	2 (20)	5 (71)	3 (38)
	6-month	8 (32)	2 (20)	3 (43)	3 (38)
	12-month	8 (32)	2 (20)	2 (29)	4 (50)
**Remission**					
	Posttreatment	12 (48)	4 (40)	6 (86)	2 (25)
	3-month	11 (44)	4 (40)	5 (71)	2 (25)
	6-month	10 (40)	3 (30)	5 (71)	2 (25)
	12-month	10 (40)	3 (30)	5 (71)	2 (25)

^a^OCD: obsessive-compulsive disorder.

^b^PD: panic disorder.

^c^SAD: social anxiety disorder.

### Cost-Effectiveness

**Figure 3:**
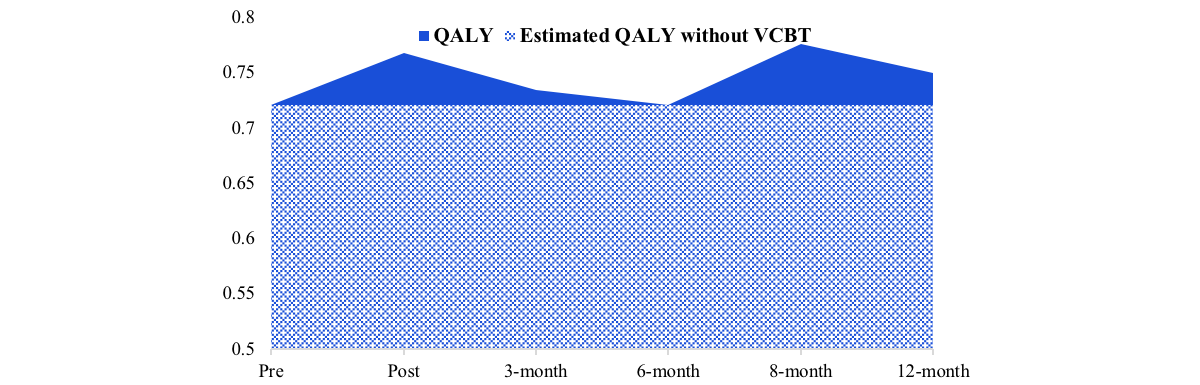
The quality-adjusted life years (QALYs) observed at follow-up and QALY in complete cases. Note: Estimated QALYs was calculated in terms of effective value without videoconference-delivered cognitive behavioral therapy at each time point as a baseline, with the area under the curve assuming no change. Increased QALY was calculated as the difference between the measured utility value and the estimated QALY.

Table 4 shows the EQ-5D-5L index for each end point. The 1-year converted QALY score from baseline to 12 months posttreatment was 0.7469 (SE 0.0353, 95% CI 0.6728-0.821), and the change between baseline and 12-month follow-up assessment was 0.0379 (SE 0.01; Table 5). Figure 3 shows the QALY calculated from the EQ-5D-5L between baseline and 12-month follow-up assessment. There was a significant increase of 0.038 (95% CI 0.0085-0.0674, *P*=.02) in complete cases. The results on the data supplemented with missing values are shown in Table 6. The WTP threshold was ¥189,500 because the 0.0379 score in the QALYs increased after the intervention. The health care costs including the VCBT accounted for the CBT health care costs (¥56,000-¥76,000), and annual licensing fees per patient for the videoconferencing system (¥4800-¥5960) was ¥60,800 to ¥81,960 (Table 7). Thus, we concluded that the VCBT was a cost-effective intervention because VCBT costs were below the threshold set for the cost-effectiveness analysis.

**Table 4:** EuroQol 5-Dimension 5-Level index each end point.

Characteristics		n	Mean	SD	SE
**Complete case**					
	Baseline	25	0.7206	0.14	—
	Posttreatment	25	0.7677	0.20	—
	3-month	25	0.7350	0.17	—
	6-month	22	0.7207	0.24	—
	8-month	20	0.7760	0.15	—
	12-month	23	0.7503	0.15	—
**LOCF^a^**					
	6-month	25	0.7342	0.23	—
	8-month	25	0.7669	0.15	—
	12-month	25	0.7530	0.15	—
**MICE^b^**					
	6-month	25	0.7075	—	0.05
	8-month	25	0.7651	—	0.03
	12-month	25	0.7564	—	0.03

^a^LOCF: last observation carried forward.

^b^MICE: multivariate imputation by chained equations.

**Table 5:** Paired *t* test results on change of quality-adjusted life years at 12 months after baseline.

Characteristics	n	Mean	SE	95% CI	*P* value
Complete cases	19	0.0379	0.01	0.0085-0.0674	.02
LOCF^a^	25	0.0214	0.01	0.0067-0.0495	.13
MICE^b^	25	0.0187	0.01	0.0093-0.0466	.19

^a^LOCF: last observation carried forward.

^b^MICE: multivariate imputation by chained equations.

**Table 6:** Quality-adjusted life years at 12 months after videoconference-delivered cognitive behavioral therapy.

Characteristics	n	Mean	SE	95% CI
Complete cases	19	0.7469	0.04	0.6728-0.8210
LOCF^a^	25	0.7420	0.03	0.6839-0.8001
MICE^b^	25	0.8343	0.04	0.7565-0.9121

^a^LOCF: last observation carried forward.

^b^MICE: multivariate imputation by chained equations.

**Table 7:** Results of a cost-utility analysis.

Characteristics		Value
**Cost for each service per patient (¥)**		
	CBT^a^ by a nurse	¥56,000
	CBT by a medical doctor	¥76,000
	Videoconferencing in Webex	¥5960
	Videoconferencing in Curon	¥4800
	Total cost	¥60,800-¥81,960
**QALY^b^**		
	Complete case	0.7469
	LOCF^c^	0.742
	MICE^d^	0.8343
**Incremental benefit, QALY gain**		
	Complete case	0.0379
	LOCF	0.0214
	MICE	0.0187
**Incremental cost-effectiveness ratio per QALY**		
	Complete case	¥1,604,222 to ¥2,162,533
	LOCF	¥2,841,122 to ¥3,829,907
	MICE	¥3,251,337 to ¥4,382,888
**Willingness to pay =** **¥5 million per QALY**		
	Complete case	¥189,500
	LOCF	¥107,000
	MICE	¥93,500

^a^CBT: cognitive behavioral therapy.

^b^QALY: quality adjusted life year.

^c^LOCF: last observation carried forward.

^d^MICE: multivariate imputation by chained equations.

## Discussion

### Principal Findings

We investigated the long-term effectiveness and cost-effectiveness of VCBT in 25 patients with OCD, PD, or SAD in a 12-month observational study. The principal symptomology of OCD, PD, and SAD significantly decreased and the QALY significantly improved. The therapeutic response rate was 32% (8/25) and remission rate was 40% (10/25) at the 12-month postintervention follow-up assessment. The total cost of providing VCBT was ¥60,800 to ¥81,960 per patient; in contrast, the threshold using WTP was ¥189,500. Therefore, our results suggested that VCBT was a cost-effective intervention for this sample of patients with OCD, PD, or SAD in Japan.

### Long-Term Effectiveness of Videoconference-Delivered Cognitive Behavioral Therapy

In a previous study on VCBT provided to 10 adult OCD patients, 2 patient scores were below the Y-BOCS cutoff (<14) after treatment, but just one patient was below the cutoff 3 months later [10]. There was a trend toward increased OCD symptoms at 3 months’ postintervention [10]. A randomized controlled trial (RCT) of VCBT provided to OCD patients aged 7 to 16 years indicated that continued improvement was observed in the symptoms until 3 months after treatment [35]. In that study [35], one of the two patients who evidenced remission before and after the treatment was still in remission 6 months’ postintervention, whereas the other patient presented worse symptoms. This study provides observational results from the end point of the VCBT for 12 months, which extends the findings of previous research. In other words, as the amount of time after the intervention increased, OCD symptoms apparently increased and the proportion of remissions apparently decreased from 40% (4/10) immediately after VCBT to 30% (3/10) at 3 months later and 20% (2/10) at the 6-month and 12-month follow-up assessments. The results of this study are consistent with previous studies that the remission rate decreases with time [7,30]. When patient symptoms increase, they might access a self-help program or attend regular support sessions to help prevent symptom relapse [36]. In a survey of adolescents with OCD, satisfaction with support sessions was universal [37].

In a study that provided VCBT to 11 adult PD patients, 82% (9/11) had improved symptoms after the intervention and 91% (10/11) had improved symptoms after 6 months and no panic attacks [38]. This study extended the examination of the long-term efficacy of VCBT in patients with PD to 12 months and found that it was effective for 85% (6/7) after treatment, and it held steady at 71% (5/7) after 3, 6, and 12 months. However, although VCBT has demonstrated its long-term efficacy, there is some indication that panic symptoms might relapse over time [25].

In a study of VCBT in 24 adult patients with SAD, 54% (13/24) experienced remission after treatment, and symptoms that had decreased were maintained at that lower level 6 months later [39]. Our results were similar to that study in that the patients who achieved remission after VCBT seemed to continue in remission for 6 or 12 months (both 2/8, 25%). The same two patients exhibited remission at any point during the 12 months. We gradually lost contact during the observation period with 2 of the 4 patients who had exhibited remission after the VCBT. Therefore, when interpreting the long-term symptom-improving effects, it might be important to consider the course of remission rather than the overall remission rate during the study period.

### Cost-Effectiveness of Videoconference-Delivered Cognitive Behavioral Therapy

Several studies have reported that internet-delivered cognitive behavioral therapy (ICBT) provided to patients with depression saved on direct medical costs more than providing just the usual care [40,41]. In an RCT conducted in Spain [41], providing ICBT to patients with depression was more cost-effective than 12 months of treatment restricted to usual care: €6381 for therapist-guided ICBT and €11,390 for nonguided ICBT. On the other hand, an RCT of ICBT aimed at preventing recurrent depression found that the average cost after 24 months was not significantly different between the ICBT group ($8298) and the usual care group ($7296) [42]. A study of face-to-face CBT in 469 participants with depression suggested that the incremental cost-effectiveness ratio was £5374 per QALY gain [43], below the threshold £20,000 to £30,000 at NICE [34]. That study’s result was consistent with our result: ¥1,604,222 to ¥2,162,533 per QALY gain (£11,459 to £15,447; calculated as ¥100=£140), below the threshold of ¥5 million in Japan [29]. Hence, CBT for depression and anxiety disorders was cost-effective whether it was face-to-face or internet intervention, with or without videoconferencing.

This study provides the world’s first empirical knowledge about the cost-effectiveness of VCBT. VCBT costs totaled ¥60,800 to ¥81,960, which was far below the ¥189,500 threshold based on WTP calculated using the QALY. In other words, under the Japanese insurance system in 2018 [28], VCBT was a cost-effective treatment approach. We determined that ¥100 was approximately $110, €120, and £140. The VCBT costs were then determined to be $553 to $745, €507 to €683, and £434 to £585 and the threshold of WTP was $1723, €1579, £1354.

### Limitations and Future Research

This study has some limitations. First, there was no statistical control over the relationship between VCBT and pharmacological therapy during our previous trial and this follow-up study. Studies have suggested that combining therapeutic approaches with drug therapy is particularly effective in panic disorder prognoses [44]. Future studies should include a controlled design that accounts for drug therapy and combination therapy. Second, we did not account for the effects of support provided to the participants during the observation period after the VCBT. Patients who continue to use antidepressants after remission were known to have a lower recurrence rate than those who discontinued prematurely [45]. Third, there was no usual care group to contrast with the VCBT group as a control in the cost-effectiveness analysis. We examined the cost-effectiveness of VCBT based on a white paper on the health care costs of patients with anxiety disorders in Japan [28], and, therefore, future research should employ actual observations and data. Fourth, during some observation periods (eg, 6 months or 12 months posttreatment), we lacked data on participants who exhibited significant symptom improvements immediately after treatment. Therefore, the results should be interpreted with caution. Fifth, a small sample size was used in this study, and there was no comparison group. Future studies should use a large sample and employ RCTs. Sixth, participants recruited in this study tended to be living in their own local areas far from our hospital without having face-to-face CBT, and they had relatively long duration of untreated illness before CBT. In future, VCBT cost-effectiveness studies for patients at the early onset stage of the disorders in primary care settings will be required.

### Conclusion

Our results suggest that VCBT for patients with OCD, PD, and SAD was effective in improving symptoms over 12 months and was a cost-effective approach in Japan.

## Abbreviations

ANOVA: analysis of variance
CBT: cognitive behavioral therapy
EQ-5D-5L: EuroQol 5-Dimension 5-Level
GAD-7: Generalized Anxiety Disorder–7
ICBT: internet-delivered cognitive behavioral therapy
ICER: incremental cost-effectiveness ratio
LOCF: last observation carried forward
LSAS: Liebowitz Social Anxiety Scale
MICE: multivariate imputation by chained equations
NICE: National Institute for Health and Care Excellence
OCD: obsessive-complusive disorder
PD: panic disorder
PDSS: Panic Disorder Severity Scale
PHQ-9: Patient Health Questionnaire–9
QALY: quality-adjusted life year
RCT: randomized controlled trial
SAD: social anxiety disorder
VCBT: videoconference-delivered cognitive behavioral therapy
WTP: willingness-to-pay
Y-BOCS: Yale-Brown Obsessive-Compulsive Scale
